# Evaluation of the HANDOC Score and the 2023 International Society of Cardiovascular Infectious Diseases and European Society of Cardiology Duke Clinical Criteria for the Diagnosis of Infective Endocarditis Among Patients With Streptococcal Bacteremia

**DOI:** 10.1093/cid/ciae315

**Published:** 2024-06-06

**Authors:** Nicolas Fourré, Virgile Zimmermann, Laurence Senn, Pierre Monney, Georgios Tzimas, Giorgia Caruana, Piergiorgio Tozzi, Matthias Kirsch, Benoit Guery, Matthaios Papadimitriou-Olivgeris

**Affiliations:** Infectious Diseases Service, Lausanne University Hospital and University of Lausanne, Lausanne, Switzerland; Infectious Diseases Service, Lausanne University Hospital and University of Lausanne, Lausanne, Switzerland; Infectious Diseases Service, Lausanne University Hospital and University of Lausanne, Lausanne, Switzerland; Infection Prevention and Control Unit, Lausanne University Hospital and University of Lausanne, Lausanne, Switzerland; Department of Cardiology, Lausanne University Hospital and University of Lausanne, Lausanne, Switzerland; Department of Cardiology, Lausanne University Hospital and University of Lausanne, Lausanne, Switzerland; Institute of Microbiology, Lausanne University Hospital and University of Lausanne, Lausanne, Switzerland; Department of Cardiac Surgery, Lausanne University Hospital and University of Lausanne, Lausanne, Switzerland; Department of Cardiac Surgery, Lausanne University Hospital and University of Lausanne, Lausanne, Switzerland; Infectious Diseases Service, Lausanne University Hospital and University of Lausanne, Lausanne, Switzerland; Infectious Diseases Service, Lausanne University Hospital and University of Lausanne, Lausanne, Switzerland; Infection Prevention and Control Unit, Lausanne University Hospital and University of Lausanne, Lausanne, Switzerland; Infectious Diseases Service, Cantonal Hospital of Sion and Institut Central des Hôpitaux, Sion, Switzerland

**Keywords:** streptococci, Duke criteria, infective endocarditis, sepsis, bloodstream infection

## Abstract

**Background:**

Streptococci are a common cause of infective endocarditis (IE). We aimed to evaluate the performance of the HANDOC score to identify patients at high risk for IE and the Duke clinical criteria of the European Society of Cardiology (ESC; 2015 and 2023 versions) and the 2023 version from the International Society of Cardiovascular Infectious Diseases (ISCVID) in diagnosing IE among patients with streptococcal bacteremia.

**Methods:**

This retrospective study included adult patients with streptococcal bacteremia hospitalized at Lausanne University Hospital. Episodes were classified as IE by the Endocarditis Team. A HANDOC score >2 classified patients as high risk for IE.

**Results:**

Among 851 episodes with streptococcal bacteremia, IE was diagnosed in 171 episodes (20%). Among 607 episodes with non-β-hemolytic streptococci, 213 (35%) had HANDOC scores >2 points; 132 (22%) had IE. The sensitivity of the HANDOC score to identify episodes at high risk for IE was 95% (95% confidence interval [CI], 90%–98%), the specificity 82% (95% CI, 78%–85%), and the negative predictive value (NPV) 98% (95% CI, 96%–99%). 2015 Duke-ESC, 2023 Duke-ISCVID, and 2023 Duke-ESC clinical criteria classified 114 (13%), 145 (17%), and 126 (15%) episodes as definite IE, respectively. Sensitivity (95% CI) for the 2015 Duke-ESC, 2023 Duke-ISCVID, and 2023 Duke-ESC clinical criteria was calculated at 65% (57%–72%), 81% (74%–86%), and 73% (65%–79%), respectively, with specificity (95% CI) at 100% (98%–100%), 99% (98%–100%), and 99% (98%–100%), respectively.

**Conclusions:**

The HANDOC score showed an excellent NPV to identify episodes at high risk for IE. Among the different versions of the Duke criteria, the 2023 Duke-ISCVID version fared better for the diagnosis of IE among streptococcal bacteremia.

## Teaser

Infective endocarditis was diagnosed in 20% of 851 episodes of streptococcal bacteremia. Sensitivity for HANDOC score >2 points, 2015 Duke-ESC, 2023 Duke-ISCVID, and 2023 Duke-ESC clinical criteria for diagnosing infective endocarditis was 95%, 65%, 81%, and 73%, respectively.

Streptococci rank among the most common culprits of infective endocarditis (IE), trailing only *Staphylococcus aureus* in frequency [1, 2]. Nevertheless, the risk of IE varies among different streptococcal species, with those belonging to the *Streptococcus mutans*, *Streptococcus sanguinis*, *Streptococcus bovis*, and *Streptococcus mitis* groups exhibiting the highest IE risk. In contrast, *Streptococcus pneumoniae* and β-hemolytic streptococci pose a lower risk [3–6]. This discrepancy led to the inclusion of a new recommendation in the 2023 European Society of Cardiology (ESC) guidelines for performing echocardiography for bacteremia caused by certain streptococcal species [7].

Despite this, uncertainty persists regarding the necessity of universal echocardiography screening for IE in patients with streptococcal bacteremia. The question remains whether these screenings should be reserved for high-risk individuals. Notably, a screening test, known as the HANDOC score, was developed specifically for patients with bacteremia caused by non-β-hemolytic streptococci to identify those at a heightened risk of IE [8]. The HANDOC score, composed of 6 variables (heart murmur or valve disease [H]; aetiology with the groups of *S mutans*, *S bovis*, *S sanguinis*, or *S anginosus* [A]; number of positive blood cultures ≥2 [N]; duration of symptoms ≥7 days [D]; only 1 species growing in blood cultures [O]; and community-acquired infection [C]), demonstrated high sensitivity and specificity in the initial study and 2 subsequent validation studies [8–10]. However, both studies had several limitations. First, the number of evaluated patients was low (223, 152, and 62 patients). Second, the reference standard was definite IE by the 2015 ESC Duke criteria, which have been shown to have a less than optimal sensitivity and specificity [2, 11–13], and exclusion of patients not undergoing transesophageal echocardiography (TEE) or those clinically treated for IE without meeting the 2015 Duke-ESC criteria for definite IE [8, 9–10].

Nonetheless, streptococci belonging to *S bovis* group and viridans streptococci (with the exception of *S pneumoniae*) have been consistently recognized as typical pathogens in all versions of the Duke criteria, including the 2023 ESC and International Society for Cardiovascular Infectious Diseases (ISCVID) Duke criteria [7, 14–16]. Moreover, the 2023 Duke-ISCVID criteria expanded the list of typical pathogens to include *Streptococcus agalactiae* and *Streptococcus dysgalactiae*, based on studies categorizing these pathogens as having a moderate risk for IE [4–6], and demonstrated improved performance compared to the 2015 Duke-ESC in various studies including different populations, such as patients with suspected IE, patients with bacteremia, or only those without IE [2, 11–13, 17, 18]. Additionally, Lindberg et al assessed the specific impact of changes in streptococcal species classification, showing that the inclusion of *S dysgalactiae* as a typical microorganism for IE led to a reclassification of most episodes from the rejected to the possible IE category, despite the low risk of IE [17].

Our study aims to assess the performance of the HANDOC score for identifying patients at high risk for IE and different versions of the Duke clinical criteria (2015 Duke-ESC, 2023 Duke-ISCVID, and 2023 Duke-ESC) for diagnosing IE in patients with streptococcal bacteremia.

## METHODS

This retrospective study was conducted at Lausanne University Hospital in Switzerland from January 2014 to March 2023. The study integrated data from 2 separate cohorts: the bacteremia cohort (January 2015 to December 2021) and the cohort comprised of patients with suspected IE (January 2014 to March 2023; suspicion of IE was defined as blood cultures drawn and echocardiography performed specifically for the research of IE). Approval for the study was granted by the ethics committee of the Canton of Vaud (CER-VD 2021–02516, CER-VD 2017-02137).

Inclusion criteria were adult patients (≥18 years old) and presence of at least 1 blood culture for *Streptococcus* spp (database of the microbiology laboratory). Exclusion criteria consisted of patients who had formally declined the use of their data, cases with incomplete medical records (including patients transferred to other hospitals at the onset of infection without follow-up data), and instances where the isolated *Streptococcus* spp was considered to be a contaminant.

Demographic (age, sex), clinical, imaging, microbiological, surgical, and pathological data were retrieved from patients’ electronic health records. Blood cultures were incubated using the BacT/ALERT System (bioMérieux, Marcy l’Etoile, France). Species identification was performed using matrix-assisted laser desorption-ionization–time of flight mass spectrometry (Bruker Daltonics, Bremen, Germany).

In our institution, the infectious diseases (ID) consultants received notifications regarding patients with positive blood cultures for streptococci, but the decision to conduct an ID consultation was left to the discretion of the consultant [19]. However, for all patients with suspected IE, an ID consultation was mandatory. Follow-up blood cultures until sterilization were performed in patients suspected of having IE. In January 2018, an Endocarditis Team was established, and since then, the diagnosis of IE (considered the reference standard) has been determined by the Endocarditis Team at day 60, based on clinical, laboratory, microbiological, imaging, surgical, and histopathological findings. For cases from 2014 to December 2017, and for cases from 2018 to 2023 not evaluated during the Endocarditis Team's weekly meetings, an informal Endocarditis Team, consisting of an ID consultant (M. P.-O.) and a cardiologist (P. M.), served the role of adjudicating IE cases as the reference standard. Both expert clinicians have been part of the Endocarditis Team since January 2018. Cases were classified as rejected, possible, or definite IE according to the 3 versions of the Duke clinical criteria (2015 Duke-ESC [14], 2023 Duke-ISCVID [15], and 2023 Duke-ESC [7]). Additionally, cases were characterized as high risk (>2 points) or low risk (≤2 points) for IE using the HANDOC score [8]. Two analyses were conducted: 1 encompassing only the intended population of non-β-hemolytic streptococci, and a second one that included all episodes of streptococcal bacteremia, with *S pneumoniae* and β-hemolytic streptococci (*S pyogenes*, *S agalactiae*, and *S dysgalactiae*) being adjudicated −1 point in the Aetiology (A) component of the HANDOC score.

The date of collection of the first positive blood culture was defined as infection onset. A new episode was included if >30 days had elapsed since the cessation of antibiotic treatment for the initial bacteremia. The classification of bacteremia cases as community, healthcare associated, or nosocomial followed the criteria established by Friedman et al [20]. The determination of the infection site was based on the assessment by the ID consultant, taking into account clinical, radiological, microbiological, and operative findings.

SPSS version 26.0 (SPSS, Chicago, Illinois) were used for data analyses. Categorical variables were analyzed using the χ^2^ or Fisher exact test and continuous variables with Mann-Whitney *U* test. For the 2 versions of the HANDOC score, the performance was calculated by measuring the level of agreement between cases at high risk for IE (>2 points) and the diagnoses determined by the Endocarditis Team. The efficacy of each version of the Duke clinical criteria was evaluated by measuring the level of agreement between definite IE cases and the diagnoses determined by the Endocarditis Team. Sensitivity, specificity, positive predictive value (PPV), negative predictive value (NPV), and accuracy were calculated with 95% confidence intervals (CIs). The performance of 2 versions of HANDOC was also assessed, with the reference standard being the definite IE by each version of the Duke criteria. All tests were 2-tailed, and a significance level of *P* < .05 was applied.

## RESULTS

**Figure 1.**
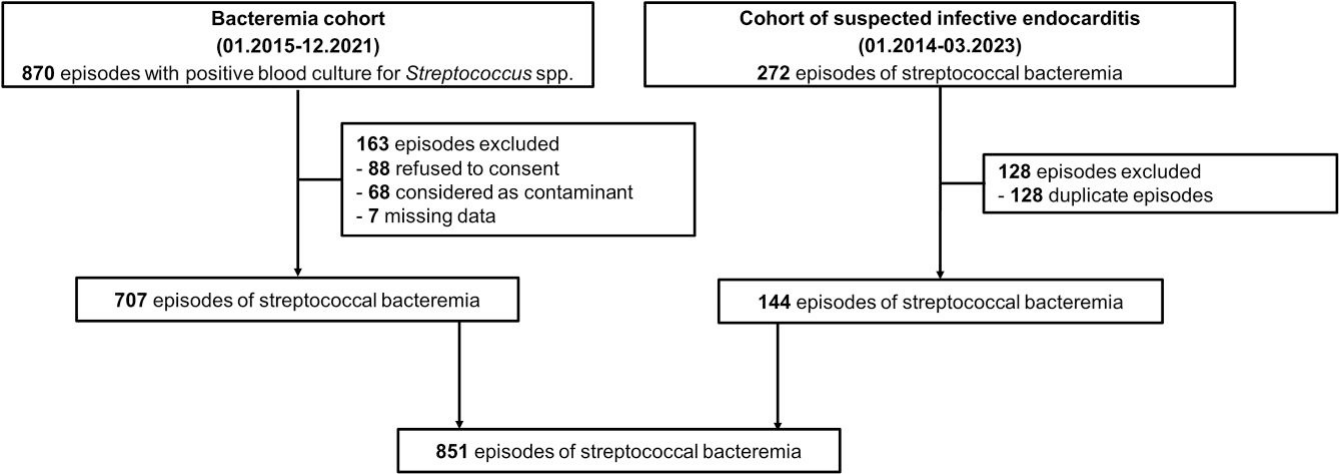
Flowchart of included episodes.

Among the 1140 episodes with positive blood cultures for streptococci, 851 episodes were included (Figure 1). Streptococci from the *S mitis* group (excluding *S pneumoniae*) were the most frequently isolated (325 [38%]), followed by *S anginosus* group (131 [15%]) and *S agalactiae* (94 [11%]) (Table 1). A total of 607 (71%) episodes exhibited bacteremia caused by non-β-hemolytic streptococci.

**Table 1.** Comparison of Episodes With or Without Infective Endocarditis Among 851 Episodes With Streptococcal Bacteremia

Characteristic	No Infective Endocarditis (n = 680)		Infective Endocarditis (n = 171)		*P* Value
Demographics					
Male sex	447	(66)	134	(78)	.001
Age, y, median (IQR)	65	(51–76)	67	(54–76)	.537
Setting of infection onset					
Community	338	(50)	150	(88)	
Healthcare-associated	94	(14)	10	(6)	
Nosocomial	248	(37)	11	(6)	<.001^a^
Cardiac predisposing factors					
Intravenous drug use	26	(4)	11	(6)	.143
Rheumatic heart disease/hypertrophic cardiomyopathy	0	(0)	4	(2)	.002
Congenital disease	7	(1)	30	(18)	<.001
Prosthetic valve	19	(3)	36	(21)	<.001
Prior endocarditis	9	(1)	16	(9)	<.001
Moderate/severe valve regurgitation/stenosis	36	(5)	27	(15)	<.001
CIED	34	(5)	20	(12)	.003
Transcatheter aortic valve replacement	6	(0.9)	4	(2)	.121
Heart transplantation	2	(0.3)	0	(0)	1.000
Left ventricular assist device	3	(0.4)	1	(0.6)	1.000
Heart murmur	60	(9)	113	(66)	<.001
Microbiological data					
≥2 blood cultures positive (initial blood cultures)	353	(52)	163	(95)	<.001
Species					
*Streptococcus pneumoniae*	36	(5)	2	(1)	.020
β-hemolytic streptococci					
*S pyogenes*	51	(8)	7	(4)	.128
*S agalactiae*	79	(12)	15	(9)	.340
*S dysgalactiae*	52	(8)	8	(5)	.241
*S anginosus* group	124	(18)	9	(5)	<.001
*S bovis* group	31	(5)	41	(24)	<.001
*S mitis* group^a^	262	(39)	63	(37)	.725
*S mutans* group	2	(0.3)	8	(5)	<.001
*S salivarius* group	71	(10)	5	(3)	.001
*S sanguinis* group	15	(2)	13	(8)	.001
Polymicrobial bloodstream infection	212	(31)	10	(6)	<.001
Persistent streptococcal bacteremia (≥48 h)	2	(0.3)	7	(4)	<.001
Infection data					
Systemic symptoms	636	(94)	168	(98)	.014
Duration of systemic symptoms, d, median (IQR)	1	(1–2)	7	(3–24)	<.001
Fever	592	(87)	144	(84)	.320
Sepsis	239	(35)	58	(34)	.788
Septic shock	98	(14)	20	(12)	.389
Vascular phenomena (major arterial emboli, septic pulmonary infarcts, mycotic aneurysm, intracranial hemorrhage, conjunctival hemorrhages, and Janeway lesions)	29	(4)	102	(60)	<.001
Cerebral abscess	1	(0.1)	1	(0.6)	.362
Immunological phenomena	3	(0.4)	15	(9)	<.001
Type of infection					
Unknown focus	153	(23)	0	(0)	<.001
Catheter-related	21	(3)	0	(0)	.012
Lower respiratory tract infection	85	(13)	1	(0.6)	<.001
Urinary tract infection	22	(3)	0	(0)	.012
Abdominal infection	148	(22)	1	(0.6)	<.001
Skin and soft tissue infection	113	(17)	3	(2)	<.001
Bone and joint infection	62	(9)	28	(16)	.008
Septic arthritis	23	(3)	12	(7)	.049
Spondylodiscitis	7	(1)	12	(7)	<.001
Other focus	103	(15)	3	(2)	<.001
Imaging data					
Positive echocardiography (either TTE or TEE) for vegetation, perforation, dehiscence of prothesis, abscess, aneurysm, pseudoaneurysm, fistula	1	(0.1)	127	(74)	<.001
Abnormal metabolic activity in ^18^F-FDG PET/CT	0	(0)	9	(5)	<.001
Abnormal metabolic activity in ^18^F-FDG PET/CT in native valve or CIED lead	0	(0)	4	(2)	.002
Abnormal metabolic activity in ^18^F-FDG PET/CT in prosthetic valve	0	(0)	5	(3)	<.001
Positive cardiac CT for vegetation, perforation, dehiscence of prothesis, abscess, aneurysm, pseudoaneurysm, fistula	1	(0.1)	8	(5)	<.001
Types of cardiac lesion					
Vegetation	0	(0)	125	(73)	<.001
Abscess	0	(0)	34	(20)	<.001
Perforation, dehiscence of prothesis	0	(0)	39	(23)	<.001
Aneurysm, pseudoaneurysm, fistula	1	(0.1)	9	(5)	<.001
Leaflet thickening	7	(1)	41	(24)	<.001
Significant new valvular regurgitation	6	(0.9)	60	(35)	<.001
Data on surgery/CIED extraction/histopathology					
Valve surgery performed	2	(0.3)	92	(54)	<.001
Macroscopic evidence of IE by inspection	0	(0)	85	(50)	<.001
CIED extraction (among 54 episodes with CIED)	2	(6)	5	(25)	.086
Autopsy performed	4	(0.6)	2	(1.2)	.347
Duke pathological criterion	0	(0)	65	(38)	<.001

Data are depicted as No. (%) unless otherwise indicated.

Abbreviations: ^18^F-FDG PET/CT, ^18^F-fluorodeoxyglucose positron emission tomography/computed tomography; CIED, cardiac implantable electronic device; CT, computed tomography; IE, infective endocarditis; IQR, interquartile range; TEE, transesophageal echocardiography; TTE, transthoracic echocardiography.

^a^Comparison between nosocomial bacteremias and both community and healthcare associated.

Transthoracic echocardiography (TTE), TEE, ^18^F-fluorodeoxyglucose positron emission tomography/computed tomography (^18^F-FDG PET/CT), and cardiac computed tomography were performed in 447 (53%), 220 (26%), 48 (6%), and 15 (2%) episodes, respectively, with 471 (55%) episodes undergoing at least 1 type of cardiac imaging. ID consultation was provided in 684 (80%) episodes.

One hundred seventy-one (20%) episodes were diagnosed with IE by the Endocarditis Team, with 132 (77%) involving native valves, 35 (20%) prosthetic valves, 6 (4%) cardiac implantable electronic device (CIED) lead, and 4 (2%) other intracardiac structures. Four episodes presented with recurrence of bacteremia and IE with the same streptococcal species within 120 days of the initial episode (Supplementary Table 1).

Among the 680 episodes without IE, other sources of bacteremia included bacteremia of unknown focus (153 [18%]), abdominal (149 [18%]), skin and soft tissue (116 [14%]), osteoarticular (90 [11%]), lower respiratory tract (86 [10%]), catheter-related (21 [2%]), and other types of infection (128 [15%]). Nine (1%) episodes presented with a recurrence of bacteremia with the same streptococcal species within 120 days of the initial episode; in every subsequent episode, IE was excluded and a clear focus of infection was diagnosed (Supplementary Table 1). Death occurred within 120 days in 128 episodes (19%); in none of these episodes was the cause of death a recurrence of IE.

The prevalence of IE among 707 episodes from the bacteremia cohort only is presented in Supplementary Table 2; IE prevalence was considered high (>10%) among the *S mutans* group (75%), *S sanguinis* group (46%), *S bovis* group (43%), and *S mitis* group (19%).

Among all streptococcal bacteremias, 265 (31%) were categorized as high risk (>2 points) by the HANDOC score (Table 2). Among 607 episodes with non-β-hemolytic streptococci, 139 (23%) were diagnosed with IE; 213 (35%) had HANDOC score exceeding 2 points. The sensitivity of the HANDOC score was 95% (95% CI, 90%–98%) and 91% (95% CI, 85%–95%) for non-β-hemolytic and all streptococci, respectively, with specificity at 82% (95% CI, 78%–85%) and 83% (95% CI, 80%–86%), respectively (Table 3). The NPV of the HANDOC score was 98% (95% CI, 96%–99%) and 97% (95% CI, 96%–98%) for non-β-hemolytic and all streptococci, respectively. When applied only in the 707 episodes from the bacteremia cohort, HANDOC score had an NPV of 99% (95% CI, 97%–99%) and 98% (95% CI, 97%–98%) for non-β-hemolytic and all streptococci, respectively (Table 4). When the reference standard was definite IE by each version of the Duke criteria, NPV was ≥98% for both versions of the HANDOC score (Supplementary Table 3).

**Table 2.** Classifications Based on the 2 Versions of the HANDOC Score and 3 Versions of the Duke Clinical Criteria

Criteria	No Infective Endocarditis		Infective Endocarditis		*P* Value
HANDOC score (all streptococci)	n = 680		n = 171		
HANDOC-H (heart murmur or valvular disease, +1 point)	111	(16)	133	(78)	<.001
HANDOC-A (aetiology)					
–1 point^a^	339	(50)	41	(24)	
0 points	293	(43)	68	(40)	
+1 point	48	(7)	57	(36)	<.001^b^
HANDOC-N (number of cultures, +1 point)	353	(52)	163	(95)	<.001
HANDOC-D (duration of symptoms, +1 point)	33	(5)	95	(56)	<.001
HANDOC-O (only 1 species, +1 point)	468	(69)	161	(94)	<.001
HANDOC-C (community-acquired, +1 point)	338	(50)	150	(88)	<.001
Final HANDOC score (points)	2	(1–2)	4	(3–5)	<.001
HANDOC score >2 points	114	(17)	155	(91)	<.001
HANDOC score (only non-β-hemolytic streptococci)	n = 468		n = 139		
HANDOC-H (heart murmur or valvular disease, +1 point)	110	(24)	117	(84)	<.001
HANDOC-A (aetiology)					
–1 point	127	(27)	9	(7)	
0 points	293	(63)	68	(49)	
+1 point	48	(10)	62	(45)	<.001^b^
HANDOC-N (number of cultures, +1 point)	212	(45)	131	(94)	<.001
HANDOC-D (duration of symptoms, +1 point)	23	(5)	83	(60)	<.001
HANDOC-O (only 1 species, +1 point)	279	(60)	130	(94)	<.001
HANDOC-C (community-acquired, +1 point)	187	(40)	120	(86)	<.001
Final HANDOC score (points)	2	(1–2)	5	(4–5)	<.001
HANDOC score >2 points	81	(17)	132	(95)	<.001
Duke criteria	n = 680		n = 171		
Duke major clinical criteria					
Major imaging criterion (2015 ESC)	1	(0.1)	125	(73)	<.001
Major imaging criterion (2023 ISCVID)	7	(1)	133	(78)	<.001
Major imaging criterion (2023 ESC)	8	(1)	136	(80)	<.001
Major surgery criterion (2023 ISCVID)	0	(0)	4	(2)	.002
Major microbiological criterion (2015 ESC)	206	(30)	131	(77)	<.001
Major microbiological criterion (2023 ISCVID)	295	(43)	157	(92)	<.001
Major microbiological criterion (2023 ESC)	206	(30)	131	(77)	<.001
Duke minor clinical criteria					
Minor microbiological criterion (2015 ESC)	262	(39)	7	(4)	<.001
Minor microbiological criterion (2023 ISCVID)	306	(45)	7	(4)	<.001
Minor microbiological criterion (2023 ESC)	262	(39)	7	(4)	<.001
Minor predisposition criterion (2015 ESC)	53	(8)	77	(45)	<.001
Minor predisposition criterion (2023 ISCVID)	108	(16)	98	(57)	<.001
Minor predisposition criterion (2023 ESC)	90	(13)	96	(56)	<.001
Minor vascular criterion (2015 ESC)	29	(4)	102	(60)	<.001
Minor vascular criterion (2023 ISCVID)	29	(4)	103	(60)	<.001
Minor vascular criterion (2023 ESC)	52	(8)	115	(67)	<.001
Minor immunological criterion (all versions)	3	(0.4)	15	(9)	<.001
Minor fever criterion (all versions)	592	(87)	144	(84)	.320
Classification according to 2015 Duke-ESC clinical criteria					
Rejected	465	(68)	7	(4)	
Possible	212	(31)	53	(31)	
Definite	3	(0.4)	111	(65)	<.001
Classification according to 2023 Duke-ISCVID clinical criteria					
Rejected	360	(53)	1	(0.6)	
Possible	313	(46)	32	(19)	
Definite	7	(1)	138	(81)	<.001
Classification according to 2023 Duke-ESC clinical criteria					
Rejected	448	(66)	4	(2)	
Possible	226	(33)	43	(26)	
Definite	6	(0.9)	124	(73)	<.001

Data are depicted as No. (%) unless otherwise indicated.

Abbreviations: ESC, European Society of Cardiology; ISCVID, International Society of Cardiovascular Infectious Diseases.

^a^Inclusion of *Streptococcus pneumoniae* and β-hemolytic streptococci (*S pyogenes*, *S agalactiae*, and *S dysgalactiae*) in the group of pathogens attributed −1 point.

^b^Comparison between the species that are attributed +1 point and those with 0 or −1.

**Table 3.** Performance of the 2 Versions of the HANDOC Score in Identifying Patients at High Risk for Infective Endocarditis and the 3 Versions of the Duke Clinical Criteria for the Diagnosis of Infective Endocarditis With the Reference Standard Being the Diagnosis of Endocarditis Team

Criteria	Sensitivity, % (95% CI)	Specificity, % (95% CI)	PPV, % (95% CI)	NPV, % (95% CI)	Accuracy, % (95% CI)
HANDOC score >2 points (all streptococci)	91 (85–95)	83 (80–86)	58 (53–62)	97 (96–98)	85 (82–87)
HANDOC score >2 points (non-β-hemolytic streptococci; n = 607)	95 (90–98)	82 (78–85)	61 (56–65)	98 (96–99)	85 (82–88)
2015 Duke-ESC	65 (57–72)	100 (98–100)	97 (92–99)	92 (90–93)	93 (91–94)
2023 Duke-ISCVID	81 (74–86)	99 (98–100)	95 (90–97)	95 (94–97)	95 (94–97)
2023 Duke-ESC	73 (65–79)	99 (98–100)	95 (90–98)	93 (92–95)	94 (92–95)

Abbreviations: CI, confidence interval; ESC, European Society of Cardiology; ISCVID, International Society of Cardiovascular Infectious Diseases; NPV, negative predictive value; PPV, positive predictive value.

**Table 4.** Performance of the 2 Versions of the HANDOC Score in Identifying Patients at High Risk for Infective Endocarditis and the 3 Versions of the Duke Clinical Criteria for the Diagnosis of Infective Endocarditis Among 707 Episodes From the Bacteremia Cohort With the Reference Standard Being the Diagnosis of Endocarditis Team

Criteria	Sensitivity, % (95% CI)	Specificity, % (95% CI)	PPV, % (95% CI)	NPV, % (95% CI)	Accuracy, % (95% CI)
HANDOC score >2 points (all streptococci)	91 (82–96)	84 (81–87)	44 (39–48)	98 (97–99)	85 (82–87)
HANDOC score >2 points (non-β-hemolytic streptococci; n = 503)	93 (84–86)	83 (79–86)	48 (42–53)	99 (97–99)	84 (81–88)
2015 Duke-ESC	58 (47–69)	100 (99–100)	94 (84–98)	95 (93–96)	94 (93–96)
2023 Duke-ISCVID	73 (63–82)	99 (98–100)	91 (82–96)	96 (95–97)	96 (94–97)
2023 Duke-ESC	66 (55–76)	99 (98–100)	93 (84–97)	96 (94–97)	95 (94–97)

Abbreviations: CI, confidence interval; ESC, European Society of Cardiology; ISCVID, International Society of Cardiovascular Infectious Diseases; NPV, negative predictive value; PPV, positive predictive value.

Among 851 episodes with streptococcal bacteremia, 114 (13%), 145 (17%), and 126 (15%) episodes were classified as definite IE by the 2015 Duke-ESC, 2023 Duke-ISCVID, and 2023 Duke-ESC clinical criteria, respectively. Supplementary Table 4 depicts the 60 episodes classified as IE by the Endocarditis Team that were categorized as rejected or possible IE by the 2015 Duke-ESC clinical criteria. Thirteen had typical cardiac lesions per the 2015 Duke-ESC criteria, but bacteremia caused by a streptococcal species not considered a typical microorganism by the same version of the criteria. An additional 18 episodes had typical cardiac lesions per the 2023 Duke-ISCVID or 2023 Duke-ESC, and 20 had definite IE confirmed by histologic or macroscopic examination during surgery, CIED extraction, or autopsy. The remaining 13 episodes were considered high risk for IE (community-acquired bacteremia by typical pathogen of unknown focus, embolic events, and/or abnormal TTE without typical cardiac lesions), but did not include TEE or ^18^F-FDG PET/CT, despite being indicated, for different reasons (advanced age and comorbidities, patient refusal, contraindication, or death). Supplementary Table 5 provides information on the 9 episodes not classified as IE by the Endocarditis Team but categorized as definite IE by any version of the Duke clinical criteria. For all of these episodes, IE diagnosis was excluded, and there was no recurrence of bacteremia by the same streptococcal species in the subsequent 120 days.

Using the 2023 Duke-ISCVID, more episodes with IE achieved the major microbiological criterion (157 [92%]) compared to both ESC criteria (131 [77%]), but at the expense of more episodes without IE also meeting the major microbiological criterion (295 [43%] vs 206 [30%]) (Table 2). This led to an increase of episodes without IE being classified as possible IE by the 2023 Duke-ISCVID (46%) compared to 2015 Duke-ESC (31%) and 2023 Duke-ESC (33%) clinical criteria. Table 3 provides an overview of the performance of the different versions of the Duke clinical criteria. Sensitivity for the 2015 Duke-ESC, 2023 Duke-ISCVID, and 2023 Duke-ESC clinical criteria was calculated at 65% (95% CI, 57%–72%), 81% (95% CI, 74%–86%), and 73% (95% CI, 65%–79%), respectively, with specificity at 100% (95% CI, 98%–100%), 99% (95% CI, 98%–100%), and 99% (95% CI, 98%–100%), respectively. When applied on the 707 episodes from the bacteremia cohort, sensitivity for the 2015 Duke-ESC, 2023 Duke-ISCVID, and the 2023 Duke-ESC clinical criteria was 58% (95% CI, 47%–69%), 73% (95% CI, 63%–82%), and 66% (95% CI, 55%–76%), respectively (Table 4).

## DISCUSSION

In this combined cohort, the incidence of IE among cases of streptococcal bacteremia was 20%, and it was 12% in the bacteremia cohort, a figure consistent with findings from previous studies [4–6, 8]. The HANDOC score exhibited good performance in identifying patients at high risk for IE when applied to the intended population (non-β-hemolytic streptococci) or among all streptococci. Furthermore, we observed an improvement in sensitivity with the 2023 Duke-ISCVID and 2023 Duke-ESC compared to the 2015 Duke-ESC clinical criteria.

In line with existing literature, the prevalence of IE varies among *Streptococcus* spp, with the *S mutans* group, *S sanguinis* group, *S bovis* group, and *S mitis* group exhibiting the highest prevalence (>10%) [4–6]. This aligns with the updated 2023 ESC guidelines recommending echocardiography for bacteremia caused by certain *Streptococcus* spp. To aid clinicians in tailoring the diagnostic workup based on the presence of specific streptococcal species and other IE risk factors, the HANDOC score was introduced for assessing IE risk among patients with bacteremia caused by non-β-hemolytic streptococci [8]. In this subgroup, the HANDOC score's sensitivity was 95%, slightly lower than the 100% reported in initial and validation studies [8–10]. This discrepancy is attributed to the design of prior studies, which excluded patients without TEE or those treated as IE but not meeting the 2015 Duke-ESC definite IE criteria [8–10]. An essential finding concerning the HANDOC score was its excellent NPV of 98%, suggesting that, in the absence of clinical suspicion, echocardiography might be considered unnecessary for patients with a HANDOC score of 2 or less. While the HANDOC score included most of the characteristics associated with IE, it omitted embolic events, which are an important predictor of IE [8, 21, 22]; 60% of IE episodes had at least 1 embolic event compared to 4% among non-IE patients (*P* < .001). It is important to note that no screening test is flawless, and such predictive tools should always be used in conjunction with clinical judgment.

In the present study, we expanded the evaluation of the HANDOC score to include all streptococci, incorporating *S pneumoniae* and β-hemolytic streptococci (*S pyogenes*, *S agalactiae*, and *S dysgalactiae*) and assigning them −1 point in the Aetiology (A) variable. When applied to the entire cohort, HANDOC performed similarly to its application solely in non-β-hemolytic streptococci, suggesting that the score's scope could be broadened to encompass all streptococci.

Both the ISCVID and the ESC introduced distinct versions of the Duke criteria in 2023, reflecting significant advancements in microbiology and imaging techniques over the past decade [7, 15]. The 2023 Duke-ISCVID made substantial modifications to the microbiological criteria, including the incorporation as typical IE pathogens of *S agalactiae* and *S dysgalactiae* alongside *S bovis* group and viridans streptococci (with the exception of *S pneumoniae*) [15]. In our study, while both 2023 versions of the Duke criteria demonstrated improved sensitivity among episodes of streptococcal bacteremia compared to the 2015 Duke-ESC, the sensitivity of the 2023 Duke-ISCVID (81%) surpassed that of the 2023 Duke-ESC (73%). Although the two 2023 versions differed in other aspects, such as the major imaging criterion (new severe valvular insufficiency in 2023 Duke-ISCVID; leaflet thickening in 2023 Duke-ESC), minor predisposition criterion (moderate/severe valve regurgitation/stenosis in 2023 Duke-ISCVID; heart transplantation and left ventricular assist device in 2023 Duke-ESC), and minor vascular criterion (cerebral and splenic abscess in 2023 Duke-ISCVID; metastatic osteoarticular complication in 2023 Duke-ESC), the changes in the microbiological criterion drove the superior performance of the 2023 Duke-ISCVID [7, 15]. In a previous study on *S aureus* bacteremia evaluating different versions of the Duke criteria, both 2023 versions resulted in comparable sensitivity [18]; the fact that for streptococci, the 2023 Duke-ISCVID's sensitivity was higher than that of the 2023 Duke-ESC underscores the significance of the inclusion by the former of *S agalactiae* and *S dysgalactiae* as typical IE microorganisms. In 2023 Duke-ISCVID, 92% of IE episodes fulfilled the major microbiological criterion, compared to 77% with the 2023 Duke-ESC. However, this enhancement comes at the cost of specificity, as 46% of the episodes without IE were classified as possible IE by the 2023 Duke-ISCVID criteria compared to the other 2 versions (31% and 33%).

Our study presents several limitations. First, it is a retrospective single-center study. However, to the best of our knowledge, this is the first investigation performed to assess the various versions of the Duke criteria within a population of streptococcal bacteremia. It is noteworthy that we included cases from the cohort of suspected IE, potentially leading to an overrepresentation of cases at high risk for IE. To address this potential bias, we provided the analyses exclusively from episodes of the bacteremia cohort (Table 4, Supplementary Table 2). Additionally, 45% of patients did not undergo any cardiac imaging study. However, the fact that the majority of patients without cardiac imaging were treated for <14 days, and the absence of any case presenting an episode of IE within 120 days from the initial episode, mitigates the risk of misdiagnosed IE cases in the non-IE group. The absence of a gold standard led to the utilization of the evaluation of the Endocarditis Team as the reference standard. To mitigate this limitation, we provided a description of cases in which the aforementioned evaluation and the clinical criteria classification were discordant (Supplementary Tables 4 and 5).

In conclusion, the alterations on streptococcal species made to the major microbiological criterion in the 2023 Duke-ISCVID resulted in increased sensitivity compared to the 2015 Duke-ESC clinical criteria, surpassing that observed in the 2023 Duke-ESC. These changes had a minor impact on specificity, which experienced a slight decrease, but a higher percentage of patients without IE were classified as possible IE by the 2023 Duke-ISCVID. Among the intended population with bacteremia due to non-β-hemolytic streptococci, the HANDOC score demonstrated an excellent NPV of 98% for identifying patients at high risk for IE. Expanding the application of the score to all streptococci did not alter its performance, suggesting that its use could be extended as a screening test to all streptococci. However, it is crucial to acknowledge that every scoring system has its imperfections. Nevertheless, when employed in conjunction with clinical judgment, these predictive scores or diagnostic tools can significantly enhance physicians’ ability to provide more informed guidance for subsequent investigations.

## Supplementary Data

Supplementary materials are available at *Clinical Infectious Diseases* online. Consisting of data provided by the authors to benefit the reader, the posted materials are not copyedited and are the sole responsibility of the authors, so questions or comments should be addressed to the corresponding author.

## Supplementary Material

ciae315_Supplementary_Data

## Notes

***Author contributions*.** M. P.-O. and B. G. conceived the idea. N. F., V. Z., L. S., P. M., G. T., G. C., P. T., M. K., and M. P.-O. collected the patients’ data. M. P.-O. supervised the project, performed the analysis, interpreted the results, and wrote the manuscript. All authors contributed to manuscript revision and read and approved the submitted version.
