# Supplementary material for: Evaluation of the HANDOC Score and the 2023 International Society of Cardiovascular Infectious Diseases and European Society of Cardiology Duke Clinical Criteria for the Diagnosis of Infective Endocarditis Among Patients With Streptococcal Bacteremia
Source: Clin Infect Dis. 2024 Jun 6;79(2):434–42. doi: 10.1093/cid/ciae315 (PMC11327781; doi:10.1093/cid/ciae315)
Supplement: ciae315_Supplementary_Data [file ciae315_supplementary_data.pdf]

Supplementary Table 1. Diagnoses of episodes with recurrent bacteremia caused by the same streptococcal species within 120 days of the initial episode

	**Species**	**Diagnosis of the initial episode**	**Diagnosis of the subsequent episode**
1	*S. mitis*	Infective endocarditis	Infective endocarditis
2	*S. mitis*	Infective endocarditis	Infective endocarditis
3	*S. agalactiae*	Infective endocarditis	Infective endocarditis
4	*S. gallolyticus*	Infective endocarditis	Infective endocarditis
5	*S. agalactiae*	Skin and soft tissue infection	Skin and soft tissue infection
6	*S. agalactiae*	Skin and soft tissue infection	Skin and soft tissue infection
7	*S. agalactiae*	Skin and soft tissue infection	Skin and soft tissue infection
8	*S. mitis*	Cholangitis	Cholangitis
9	*S. anginosus*	Cholangitis	Cholangitis
10	*S. anginosus*	Cholangitis	Cholangitis
11	*S. salvarius*	Cholangitis	Cholangitis
12	*S. salvarius*	Cholangitis	Cholangitis
13	*S. anginosus*	Pulmonary abscess	Pulmonary abscess

Supplementary Table 2. Prevalence of infective endocarditis of different streptococcal species among 707 episodes from the bacteremia cohort

	No infective endocarditis (n=621)		Infective endocarditis (n=86)		Prevalence of Infective endocarditis
S. pneumoniae	29	5%	2	2%	6%
Beta-hemolytic streptococci					
S. pyogenes	47	8%	2	2%	4%
S. agalactiae	71	11%	7	8%	9%
S. dysgalactiae	48	8%	3	4%	6%
S. anginosus group	111	18%	5	6%	4%
S. bovis group	25	4%	19	22%	43%
S. mitis group[a]	262	56%	63	45%	19%
S. mutans group	2	0.3%	6	7%	75%
S. salivarius group	68	11%	2	2%	3%
S. sanguinis group	15	3%	13	9%	46%

Data are depicted as number and percentage

Supplementary Table 3. Performance of the two versions of the HANDOC score in identifying patients at high-risk for infective endocarditis with the reference standard being definite infective endocarditis by each version of the Duke criteria

	**Sensitivity % (95% CI)**	**Specificity % (95% CI)**	**PPV % (95% CI)**	**NPV % (95% CI)**	**Accuracy % (95% CI)**
	2015 Duke-ESC				
HANDOC score >2 points (all streptococci)	95 (90-98)	78 (74-81)	42(39-46)	99 (98-100)	80 (77-83)
HANDOC score >2 points (non-beta-hemolytic streptococci; n=503)	96 (90-99)	77 (73-80)	48 (44-52)	99 (97-99)	80 (77-83)
	2023 Duke-ISCVID				
HANDOC score >2 points (all streptococci)	94 (89-97)	79 (76-82)	49 (45-53)	98 (97-99)	82 (79-84)
HANDOC score >2 points (non-beta-hemolytic streptococci; n=503)	96 (90-99)	77 (73-81)	51 (47-55)	99 (97-99)	81 (78-84)
	2023 Duke-ESC				
HANDOC score >2 points (all streptococci)	96 (91-98)	79 (76-82)	47 (43-50)	99 (98-100)	82 (79-84)
HANDOC score >2 points (non-beta-hemolytic streptococci; n=503)	96 (91-99)	78 (74-82)	52 (48-56)	99 (97-99)	82 (78-85)

ESC: European Society of Cardiology; ISCVID: International Society of Cardiovascular Infectious Diseases

Supplementary Table 4. Episodes of infective endocarditis (Endocarditis Team classification) categorized as rejected or possible infective endocarditis by the 2015 Duke-ESC clinical criteria

	**Microbiologic criterion (positive bcs)**	**Imaging criterion**	**Predisposition**	**Fever**	**Vascular phenomena**	**Immunologic phenomena**	**Valve surgery**	**Surgical criterion**	**Pathological criterion**	**Other information**
1	*S. agalactiae* (2 bcs)	Significant new valvular regurgitation	Bicuspid aortic valve	Y			Y	Y	Y	
2	*S. dysgalactiae* (2 bcs)	Valve vegetation		Y	Cerebral emboli, Janeway lesions		N	N	N	
3	*S. dysgalactiae* (2 bcs)	Valve vegetation, abscess		N	Pulmonary septic emboli		N	N	N	
4	*S. gallolyticus* (2 bcs)	Small mobile element (degenerative)	CIED	Y			N	N	N	Septic arthritis; positive CIED-lead culture
5	*S. agalactiae* (3 bcs)	Valve leaflet thickening		Y			N	N	N	Septic arthritis
6	*S. mitis* (3 bcs)	Significant new valvular regurgitation	Prolapsus of mitral valve	Y			Y	Y	Y	
7	*S. sanguinis* (2 bcs)		TAVI	N	Janeway lesions		N	N	N	
8	*S. mitis* (5 bcs)	Significant new valvular regurgitation, valve leaflet thickening		Y			Y	Y	N	
9	*S. agalactiae* (4 bcs)	Valve vegetation, significant new		Y	Cerebral emboli		N	N	N	

		valvular regurgitation							
10	*S. gallolyticus* (2 bcs)	Small mobile element (degenerative)	Prolapsus of mitral valve	Y		N	N	N	
11	*S. agalactiae* (4 bcs)	Valve vegetation		Y		N	N	N	
12	*S. mutans* (2 bcs)	Small mobile element (degenerative)	Prolapsus of mitral valve	Y		N	N	N	Patient's refusal to undergo TOE
13	*S. gallolyticus* (2 bcs)		Prior IE, CIED	Y		N	N	N	Recurrence of bacteremia by same pathogen as prior episode; death before TOE
14	*S. mutans* (2 bcs)	Valve vegetation, perforation		N	Janeway lesions	Y	Y	Y	Spondylodiscitis
15	*S. mitis* (1 bcs)	Small mobile element (degenerative)		Y	Renal emboli	N	N	N	
16	*S. pneumoniae* (2 bcs)	CIED-cable lesion (degenerative)	CIED	Y		N	N	N	Septic arthritis
17	*S. gallolyticus* (4 bcs)	CIED-cable lesion (degenerative)	CIED	Y		N	N	N	Positive CIED-lead culture
18	*S. agalactiae* (2 bcs)	Valve vegetation		Y	Cerebral emboli	N	N	N	
19	*S. pyogenes* (8 bcs)	Valve vegetation	Prosthetic valve, prior IE	Y		Y	Y	Y	
20	*S. mitis* (2 bcs)		Moderate valve regurgitation	Y		N	N	N	Patient's refusal to undergo TOE; bacteremia of unknown origin

21	*S. mitis* (2 bcs)	CIED-cable lesion (degenerative)	CIED	N		N	N	N	TOE not performed due to age/comorbidities; bacteremia of unknown origin
22	*S. sanguinis* (1 bcs)	Valve vegetation, significant new valvular regurgitation		Y		Y	Y	Y	Spondylodiscitis
23	*S. mitis* (2 bcs)	Small mobile element (degenerative)		N	Pulmonary septic emboli	Y	Y	Y	
24	*S. agalactiae* (2 bcs)	Valve vegetation, abscess, perforation		Y		Y	Y	Y	Septic arthritis
25	*S. anginosus* (1 bcs)		CHD with prosthetic material	Y	Cerebral emboli	N	N	N	TOE not performed due to contraindication
26	*S. mitis* (2 bcs)	CIED-cable lesion (degenerative)	CIED, LVAD	Y		N	N	N	Bacteremia of unknown origin; persistent bacteremia[a]
27	*S. pyogenes* (3 bcs)	Valve vegetation		Y	Cerebral emboli	Y	Y	N	Spondylodiscitis
28	*S. mitis* (2 bcs)	Significant new valvular regurgitation, valve leaflet thickening	Prolapsus of mitral valve	Y		Y	Y	Y	
29	*S. gallolyticus* (2 bcs)		CIED	Y	Cerebral emboli	N	N	Y	Valve IE at autopsy
30	*S. dysgalactiae* (2 bcs)	Valve vegetation		Y		N	N	N	Septic arthritis

31	*S. dysgalactiae* (2 bcs)	Small mobile element (degenerative)		Y		N	N	N	Septic arthritis
32	*S. mitis* (1 bcs)	Significant new valvular regurgitation	Prosthetic valve	Y		Y	Y	Y	Persistent bacteremia[a]
33	*S. dysgalactiae* (2 bcs)		IVDU, prosthetic valve, prior IE	Y	Pulmonary septic emboli	N	N	N	Patient's refusal to undergo TOE or ^{18}F-FDG PET/CT
34	*S. mitis* (1 bcs)	Valve vegetation		N	Cerebral emboli	N	N	N	Spondylodiscitis
35	*S. agalactiae* (2 bcs)	Valve vegetation		Y	Splenic emboli	Y	Y	N	
36	*S. mutans* (2 bcs)	Significant new valvular regurgitation, valve leaflet thickening	Bicuspid aortic valve	Y		Y	Y	Y	
37	*S. pyogenes* (2 bcs)	Valve vegetation		Y	Cerebral emboli	N	N	N	
38	*S. agalactiae* (2 bcs)	Valve vegetation, perforation, leaflet thickening		Y	Cerebral emboli	Y	Y	N	
39	*S. mitis* (2 bcs)	Significant new valvular regurgitation, valve leaflet thickening		N		Y	Y	Y	
40	*S. agalactiae* (2 bcs)	Valve leaflet thickening	Prosthetic valve, prior IE	Y	Cerebral emboli	N	N	N	

41	*S. agalactiae* (2 bcs)	Small mobile element (degenerative)	TAVI, CIED	Y	Cerebral emboli	N	N	N	TOE not performed due to age/comorbidities; bacteremia of unknown origin
42	*S. agalactiae* (2 bcs)	Abnormal metabolic activity (^{18}F-FDG PET/CT) of native valve		Y		N	N	N	
43	*S. mitis* (3 bcs)	Valve leaflet thickening	Prosthetic valve	Y		N	N	N	
44	*S. gallolyticus* (2 bcs)		Prolapsus of mitral valve	Y		N	N	N	TOE not performed due to age/comorbidities; bacteremia of unknown origin
45	*S. gallolyticus* (2 bcs)	Valve leaflet thickening		Y		N	N	N	
46	*S. pyogenes* (2 bcs)	Valve vegetation, abscess, perforation		Y	Janeway lesions	Y	Y	N	Septic arthritis
47	*S. gallolyticus* (4 bcs)			Y	Cerebral emboli	N	N	N	Patient's refusal to undergo TOE
48	*S. gallolyticus* (2 bcs)	Small mobile element (degenerative)	Prosthetic valve	Y		N	N	N	
49	*S. mitis* (2 bcs)	Small mobile element (degenerative)	Prolapsus of mitral valve	N	Cerebral emboli	N	N	N	
50	*S. mitis* (4 bcs)		IVDU	Y		N	N	N	Patient's refusal to undergo TOE;

									bacteremia of unknown origin
51	*S. pyogenes* (2 bcs)	Valve vegetation		Y		N	N	N	
52	*S. mutans* (2 bcs)	Valve leaflet thickening	Prolapsus of mitral valve	Y		N	N	N	Spondylodiscitis
53	*S. gallolyticus* (2 bcs)	Valve leaflet thickening		Y		N	N	N	Spondylodiscitis
54	*S. salivarius* (2 bcs)	Significant new valvular regurgitation, small mobile element (degenerative)		Y		N	N	N	
55	*S. sanguinis* (2 bcs)	CIED-cable lesion (degenerative)	CIED	Y		N	N	N	TOE not performed due to age/comorbidities; bacteremia of unknown origin
56	*S. mitis* (2 bcs)			N	Arterial emboli	N	N	N	TOE not performed due to age/comorbidities; bacteremia of unknown origin
57	*S. mitis* (2 bcs)	Significant new valvular regurgitation	Moderate valve stenosis	Y		N	N	N	
58	*S. agalactiae* (2 bcs)	Valve vegetation, abscess, dehiscence	Prosthetic valve, prior IE	Y		Y	Y	Y	
59	*S. pneumoniae* (2 bcs)	Valve vegetation, significant new		Y	Cerebral emboli,	N	N	N	

		valvular regurgitation		renal emboli				
60	*S. mutans* (2 bcs)	Small mobile element (degenerative)	Y		N	N	N	TOE not performed due to age/comorbidities; bacteremia of unknown origin

^{18}F-FDG PET/CT: ^{18}F-Fluorodeoxyglucose Positron Emission Tomography/Computed Tomography; bcs: blood culture set; CIED: cardiac implantable electronic device; IE: infective endocarditis; IVDU: intravenous drug use; LVAD: left ventricular assist device; N: no; TAVI: transcatheter aortic valve implantation; TEE: transesophageal echocardiography; Y: yes

[a]for at least 48h from antimicrobial treatment initiation

Supplementary Table 5. Episodes without infective endocarditis (Endocarditis Team classification) categorized as definite infective endocarditis by either version of the Duke clinical criteria

	Microbiologic criterion (positive bcs)	Imaging criterion	Predisposition	Fever	Vascular phenomena	Immunologic phenomena	Other information
1	*S. agalactiae* (3 bcs)		Moderate valve stenosis	Y	Pulmonary septic emboli		Septic arthritis and contiguous septic deep vein thrombosis; pulmonary septic emboli attributed to septic deep vein thrombosis; TEE negative for IE; no recurrence of bacteremia in the subsequent 120 days
2	*S. anginosus* (2 bcs)		IVDU	Y	Mycotic aneurysm	Roth spots	Contiguous mycotic aneurysm (IVDU); TEE negative for IE; no recurrence of bacteremia in the subsequent 120 days
3	*S. anginosus* (2 bcs)		IVDU	Y	Mycotic aneurysm		Contiguous mycotic aneurysm (IVDU); TEE negative for IE; no recurrence of bacteremia in the subsequent 120 days
4	*S. dysgalactiae* (2 bcs)		IVDU	Y	Mycotic aneurysm		Contiguous mycotic aneurysm (IVDU); TEE negative for IE; no recurrence of bacteremia in the subsequent 120 days
5	*S. mitis* (2 bcs)	Significant new valvular regurgitation	CIED	Y			Surgery and CIED extraction at day 3 of antimicrobial treatment without macroscopic or pathological signs of IE; significant new valvular regurgitation due to chordal rupture; no recurrence of bacteremia in the subsequent 120 days
6	*S. mitis* (1 bcs)	Valve leaflet thickening	IVDU	Y	Mycotic aneurysm		Contiguous mycotic aneurysm (IVDU); TEE negative for IE; no recurrence of bacteremia in the subsequent 120 days
7	*S. gallolyticus* (2 bcs)	Valve leaflet thickening		N			Spondylodiscitis; known valve leaflet thickening; TEE negative for IE; no recurrence of bacteremia in the subsequent 120 days
8	*S. pneumoniae* (2 bcs)	Significant new valvular regurgitation, valve leaflet thickening	Bicuspid aortic valve	Y	Cerebral emboli		Bacteremia due to low-respiratory tract infection; stroke at day 10 due to atrial fibrillation; TEE negative for IE; no recurrence of bacteremia in the subsequent 120 days
9	*S. anginosus* (2 bcs)		IVDU	Y	Mycotic aneurysm		Contiguous mycotic aneurysm (IVDU); TEE negative for IE; no recurrence of bacteremia in the subsequent 120 days

bcs: blood culture set; CIED: cardiac implantable electronic device; IE: infective endocarditis; IVDU: intravenous drug use; N: no; TEE: transesophageal echocardiography; Y: yes
